# Co-release of tick-borne encephalitis virus RNA and structural proteins via virus-like particles and extracellular vesicles

**DOI:** 10.1099/jgv.0.002258

**Published:** 2026-04-29

**Authors:** Ivana Křížová, Anna Klimešová, Alžběta Dostálková, Jana Racková, Jiřina Kaufmanová, Radim Novotný, Matěj Danda, Ivana Matošević, Zdeněk Franta, Hana Tykalová, Libor Grubhoffer, Michaela Rumlová

**Affiliations:** 1 Department of Biotechnology, University of Chemistry and Technology, Prague, Technická 5, 166 28 Prague 6, Czech Republic; 2 Department of Biochemistry and Microbiology, University of Chemistry and Technology, Prague, Technická 3, 166 28 Prague 6, Czech Republic; 3 Faculty of Science, University of South Bohemia, České Budějovice, Branišovská 1760, 370 05 České Budějovice, Czech Republic; 4 Institute of Parasitology, Biology Centre,, The Czech Academy of Sciences, Branišovská 1160/31, 370 05 České Budějovice, Czech Republic

**Keywords:** C protein, extracellular vesicles, flavivirus, RNA packaging, tick-borne encephalitis virus, virus-like particles

## Abstract

Tick-borne encephalitis virus (TBEV) is a medically important flavivirus that causes severe neurological diseases in humans. The assembly of flaviviruses is initiated by the interaction between capsid (C) proteins and viral genomic RNA, yet the molecular determinants that govern RNA encapsidation remain unclear. In this study, we established a TBEV virus-like particle (VLP) system to analyse viral factors that influence viral RNA incorporation independently of productive infection. Using a reporter-containing TBEV minigenome, we investigated the contribution of UTRs to the incorporation of RNA into extracellular particles. Truncation of the 5′ UTR, 3′ UTR or both did not significantly affect the levels of minigenome RNA detected in pelleted extracellular fractions, indicating that RNA incorporation occurs largely in a non-specific manner. We further examined the role of C protein dimerization by introducing alanine substitutions into residues that form the α2–α2′ and α4–α4′ dimer interfaces. Paradoxically, these substitutions increased the levels of minigenome RNA detected in pelleted extracellular particles without altering intracellular RNA expression, indicating a complex relationship between the integrity of the C protein dimer interface and levels of extracellular viral RNA. Finally, we showed that TBEV proteins and minigenome RNA can be detected in extracellular vesicle (EV)-associated fractions under VLP-producing conditions. Using immunoaffinity purification, we demonstrate the presence of viral components in EVs, underscoring EV-associated release as a factor that complicates the analysis of flaviviral particle assembly.

## Introduction

Tick-borne encephalitis virus (TBEV) [1] is a member of the genus *Orthoflavivirus* (family *Flaviviridae*), which comprises a diverse group of arthropod-borne viruses transmitted by mosquitoes or ticks. The mosquito-borne flaviviruses include Dengue virus (DENV), Zika virus (ZIKV), Yellow fever virus (YFV) and West Nile virus (WNV) [2,3], whereas tick-borne flaviviruses comprise Powassan virus, Langat virus (LGTV), Japanese encephalitis virus (JEV) and TBEV [4,5]. TBEV is transmitted primarily by hard ticks of the genus *Ixodes*; however, infection can also occur by consuming unpasteurized dairy products [6,11]. In humans, TBEV causes tick-borne encephalitis, a disease that ranges from mild flu-like illness to severe neurological manifestations, including meningitis, encephalitis and myelitis [6,11, 12]. Although most infections are asymptomatic, symptomatic cases can lead to long-term disease or death. Disease severity varies markedly among the three major TBEV subtypes: European, Siberian and Far-Eastern, with reported mortality rates ranging from 0.5 to 2% for the European subtype to up to 40% for the Far-Eastern subtype [6,11,13]. The incidence of tick-borne encephalitis has increased in recent decades, partly due to the expansion of tick habitats associated with climate change and also changes in human activity patterns [10,14,16]. Although effective vaccines are available, treatment remains purely symptomatic, underscoring the need for a detailed understanding of the TBEV replication cycle to support the development of antiviral strategies [17,19].

Flaviviruses possess a single copy of a positive-sense RNA genome of ~11 kb, organized as a single ORF, which is flanked by 5′ and 3′ UTRs. The ORF is translated as a single polyprotein subsequently processed into three structural proteins: capsid (C), premembrane/membrane (prM) and envelope (E); and seven nonstructural proteins. Viral replication and assembly occur at the membranes of the endoplasmic reticulum (ER), where newly synthesized viral genomic RNA (gRNA) is encapsidated by the C protein to form nucleocapsids that bud into the ER lumen and acquire a lipid envelope containing prM and E proteins [20,23]. Subsequent maturation results in structural changes and the formation of infectious virions of ~50 nm in size, which are released via exocytosis [22,24, 25].

The C protein is a small (~10 kDa) basic protein that plays a central role in genome packaging. Structural studies have shown that flaviviral C proteins adopt a conserved four-helix bundle fold and form homodimers through defined α2–α2′ and α4–α4′ interfaces, generating distinct membrane-binding and RNA-binding surfaces, respectively. Recent studies on DENV, JEV and TBEV have supported a model in which the C protein interacts with the membrane and gRNA simultaneously [26,27]. Despite these structural insights, the molecular determinants that govern viral RNA encapsidation remain poorly understood. In particular, it is unclear whether genome packaging depends on specific RNA elements, such as UTRs and specific protein elements, such as NS2A [28,29], or whether it is driven primarily by non-specific electrostatic interactions. Moreover, the functional role of C protein dimerization in RNA packaging, whether dimerization precedes RNA and ER membrane binding or is instead triggered by these interactions, remains unresolved.

Virus-like particle (VLP) systems provide a powerful experimental platform to address these unresolved questions. Unlike studies based on productive infection, VLP-based approaches allow independent manipulation of viral proteins and RNA, enabling targeted analysis of genome packaging without interference of viral replication and host immune responses. In particular, the use of reporter-containing minigenomes allows the quantitative assessment of RNA incorporation into released particles, whereas site-directed mutagenesis of the C protein enables the direct evaluation of structural determinants involved in nucleocapsid formation. Therefore, such systems offer a versatile framework for investigating the specificity of C–RNA interactions and the functional role of C protein dimerization in flaviviruses.

In addition to canonical virus particle assembly and release, flaviviral proteins and RNA can be released from transfected or infected cells via extracellular vesicles (EVs) [30,31]. In general, EVs are membrane-bound vesicles, typically 30–150 nm in diameter, that mediate intercellular communication and can deliver various viral and cellular cargo [32,38]. Importantly, EVs overlap with VLPs in terms of size and density, which may complicate the interpretation of VLP-based analyses relying on conventional ultracentrifugation and density gradient separation. Therefore, consideration of EV-associated release pathways is essential for the accurate assessment of RNA packaging and particle composition in VLP systems.

Although the interaction of gRNA with the C protein and subsequent nucleocapsid formation represents the initial step of assembly, elucidating the underlying mechanism remains challenging due to the complexity of the process and the spatial and temporal overlap between RNA replication, genome packaging and C protein dimerization. In this study, we established a TBEV VLP system to analyse the molecular factors involved in viral RNA packaging and to examine the role of C protein dimerization in this process. Using a TBEV-gRNA-derived minigenome and targeted mutations in the C protein, we quantitatively assessed RNA incorporation into released particles, evaluated the contribution of untranslated gRNA regions and defined the effect of C protein dimerization on RNA incorporation. In parallel, we examined the extent to which viral proteins and RNA are co-released via EVs. This highlights an additional layer of complexity that must be considered when interpreting VLP-based analyses.

## Methods

### Preparation of expression plasmids

Three types of plasmids carrying different TBEV strain Hypr genes were prepared and used for a transfection of HEK 293 cells for the production of TBEV VLPs. These were the plasmids comprising the gene encoding a polyprotein C/prM/E, the plasmid comprising the gene encoding a protease NS2B-NS3 and the plasmid containing a TBEV-gRNA-derived minigenome purposed for packaging into VLPs [39]. The pCMV-CME plasmid, which contains a gene encoding the polyprotein, including structural proteins, i.e. C, prM and E, was obtained from Dr. Ján Štěrba, University of South Bohemia in České Budějovice, Czech Republic. In our laboratory, the plasmid was modified by adding a FLAG tag at three different sites of the TBEV C protein: downstream of arginine in position 16 (16FLAG-C), downstream of proline in position 28 (28FLAG-C) and downstream of arginine in position 91 (91FLAG-C). Following the introduction of the FLAG tag into the C protein, several amino acid residues of the C protein were substituted for alanine to prepare mutants: H2 C (M39, R40, M42, W46 and V49), H4 C (L67, T71, L74, R75, K78, R79, S82 and M85) and H2+H4 C (the combination of H2 and H4). All plasmids were constructed by EMILI mutagenesis [40] using primers listed in Table S1, available in the online Supplementary Material, and were verified by sequencing.

The gene encoding NS2B-NS3 protease was constructed by restriction cleavage and ligation into the pCMV-HA plasmid using *EcoRI*, *KpnI* and *NotI* restriction enzymes and *T4 DNA ligase*, respectively. All fragments were amplified using primers listed in Table S1, and successful insertion was verified by sequencing. To introduce GFP at the N- or C-terminus of NS2B-NS3, Gibson Assembly was applied according to the manufacturer’s protocol (Gibson Assembly^®^ Master Mix, NEB #E2611). Individual fragments have been amplified using primers listed in Table S1 and sequence-verified.

TBEV minigenome with mCherry reporter has been designed according to Osawa *et al*. [41] using the TBEV Hypr strain. Briefly, the 5′ UTR together with the fragment of the gene encoding the first 20AA of C protein, the 3′ UTR and the reporter gene mCherry have been amplified using Q5 polymerase and inserted into the pUC19 vector using the NEBuilder cloning kit according to the manufacturer’s protocol. To facilitate *in vitro* RNA transcription*,* the minigenome was flanked by a T7 promoter positioned upstream of the 5′ UTR and a *XhoI* restriction site positioned downstream of the 3′ UTR. The final construct was sequence-verified. The primers used for minigenome assembly are listed in Table S1.

The minigenome was recloned in the plasmid pCMV-HA using *SacI* and *XhoI* restriction enzymes to produce pCMV-HA-TBEV-MG. Restriction sites were introduced using primers listed in Table S1. The pCMV-HA-TBEV-MG then served as a template to generate truncated variants ∆3UTR, ∆5UTR and ∆UTRs using primers listed in Table S1. All constructs were verified by sequencing.

### Culture cells and TBEV VLP production

At 24 h before transfection, human embryonic kidney 293 cells (HEK 293 cells), grown in Dulbecco’s modified Eagle’s medium supplemented with 10% FBS and 2 mM l-glutamine, were seeded at a concentration of 3×10^5^ cells ml^−1^ to produce VLPs. HEK 293 cells were co-transfected with three plasmids, pCMV TBEV CME, pCMV TBEV NS2B-NS3 and pCMV TBEV MG, at the ratio of 2 : 1 : 2. Transfection was carried out with polyethylenimine (PEI) at the DNA:PEI ratio of 1 : 2. After 4 days, the medium containing released TBEV VLPs was harvested and processed as described below. The mock sample corresponded to non-transfected HEK 293 cells.

### Isolation and purification of extracellular material containing TBEV VLPs and EVs

Four days post-transfection, the medium containing extracellular particles, including TBEV VLPs and EVs, was harvested, filtered through a 0.45 µm membrane and treated with Turbo DNase at 37 °C for 40 min. To concentrate and isolate the extracellular particles, the medium was ultracentrifuged through a 20% (w/w) sucrose cushion at 87,000 ***g*** for 2 h in a Beckman SW28 rotor [39]. The pellet was resuspended in protein loading buffer (PLB) or PBS. For further purification, the pellet resuspended in PBS was loaded onto a linear sucrose density gradient ranging from 20 to 50% (w/v). The 1 ml gradient fractions were analysed using immunodetection.

### Isolation of EVs

HEK 293 cells were transfected with appropriate plasmids as described above. Four days later, the medium containing extracellular particles, including EVs, was harvested, filtered and ultracentrifuged through a 20% sucrose cushion. The pellet was resuspended in 0.5 ml of PBS, and EVs were specifically isolated using EasySep^™^ Human Pan-Extracellular Vesicle Positive Selection Kit according to the manufacturer’s protocol (STEMCELL Technologies). The final magnetic antibody-bead complex with bound EVs was resuspended in PBS. One portion was used to isolate total RNA, and the rest of the sample was separated using SDS-PAGE, followed by Western blot with immunodetection.

### Western blot with immunodetection

HEK 293 cells and the culture medium were harvested 4 days after transfection. Cells were washed with PBS and lysed by adding PLB (2×) (0.1 M Tris-HCl pH 6.8, 4% SDS, 20% glycerol, 0.01% bromophenol blue, 10% 2-mercaptoethanol) over the adherent cell layer. The lysate was transferred into the tubes. The medium was filtered through a 0.45 µm pore membrane, and the resulting material was ultracentrifuged through the 20% (w/w) sucrose cushion at 87,000 ***g*** for 2 h. The pellet was resuspended in PLB and separated using SDS-PAGE, followed by blotting onto a nitrocellulose membrane. The membrane was blocked for 2 h, and the respective flaviviral proteins were detected using the following antibodies: mouse antibody against the FLAG tag (MxFLAG, SCBT, Germany), rabbit antibody against the E protein (RbxE, University of South Bohemia in České Budějovice, Czech Republic) and mouse antibody against the HA tag (MxHA, Sigma, USA). The antigen–antibody complexes were detected by SuperSignal West Atto Ultimate Sensitivity Substrate (Thermo Fisher Scientific, USA) and visualized using the FUSION 7S system (Vilber Lourmat, Marne-la-Vallée, France).

### Transmission electron microscopy

The morphology of isolated extracellular particles, including VLPs, was analysed by transmission electron microscopy (TEM) of negatively stained samples. The isolated particles were placed on carbon-coated copper grids for 2 min to settle down, and excess fluid was blotted away. Then, the grids were washed twice with deionized water for 30 s and stained with 1% uranyl acetate for 40 s. After drying, the samples were analysed with a transmission electron microscope, Talos L120C G2, using 120 kV.

### Isolation of cellular RNA and RNA packaged to extracellular particles

Total RNA was isolated from HEK 293 cells 4 days after transfection using the RNeasy Mini Kit (Qiagen, Hilden, Germany) and eluted with RNase-free water. All steps were performed according to the manufacturer’s protocol, including DNase treatment. RNA packaged into the extracellular particles was isolated using the QIAamp viral RNA Mini Kit (Qiagen, Hilden, Germany) directly after harvesting, ultracentrifugation through a 20% sucrose cushion and resuspension (see above) of pelleted extracellular particles (PEPs) in PBS. The packaged RNA was eluted by the manufacturer’s buffer and treated with Turbo DNase for 40 min at 37 °C. DNase was inactivated by heating the mixture at 70 °C for 13 min.

### Detection and quantification of RNA

Isolated RNA from HEK 293 cells or PEPs was reverse transcribed into cDNA using the RevertAid First Strand cDNA Synthesis Kit (ThermoFisher Scientific), using the combination of random hexamers and Oligo(dT)_18_ primers according to the manufacturer’s protocol. The synthesized cDNA was then used as a template for quantitative PCR (qPCR). MG RNA was detected with the primers annealing to the mCherry coding sequence: mCherry-F and mCherry-R. To normalize gene expression, GAPDH was used as a reference gene: GAPDH-F and GAPDH-R. All primers are listed in Table S1. To control and normalize reverse transcription and qPCR, an internal control TATAA universal RNA spike I (TATAA Biocenter, Gothenburg, Sweden) was added to each sample before reverse transcription. The qPCR reaction was prepared by mixing 2 µl of cDNA, 2× SYTO-9 master mix, ROX as the reference dye at the final concentration of 25 nM and 1 µM primers and then performed by using a QuantStudio 5 real-time PCR system (Applied Biosystems, Waltham, MA, USA) under the following reaction conditions: 2 min at 50 °C, 2 min at 95 °C, followed by 40 cycles of 1 min at 94 °C, 30 s at 65 °C (65 °C for *mCherry*, 60 °C for Spike and 55 °C for *GAPDH*) and 30 s at 72 °C. The ΔΔCq method was applied to evaluate the level of MG RNA in the tested samples.

### Confocal fluorescence microscopy

The mScarlet-DHX15 HEK 293 cells [42] were seeded on 12 mm glass cover slips in a 24-well plate. To stain ER, ER-Tracker Blue-White DPX (Molecular Probes) was used according to the manufacturer’s instructions. At 24 h post-transfection, the cells were fixed in 4% paraformaldehyde in PBS at room temperature and imaged with a spinning disc confocal microscope (Andor, Belfast, UK) with a 100× oil objective. Colocalization analysis was done in Fiji software, and seven images were analysed.

### Protein structure visualization and modelling

TBEV C homodimer visualizations were performed using our previously determined NMR structure (PDB ID: 7YWQ) [43]. Schematics were created in PyMOL Molecular Graphics System, Version 2.5.4 (Schrödinger, LLC). The built-in ‘Builder’ tool was used to model the missing N-terminus (1–16) for visualization of FLAG insertion sites.

## Results

### Production and characterization of TBEV VLPs

**Fig. 1.**
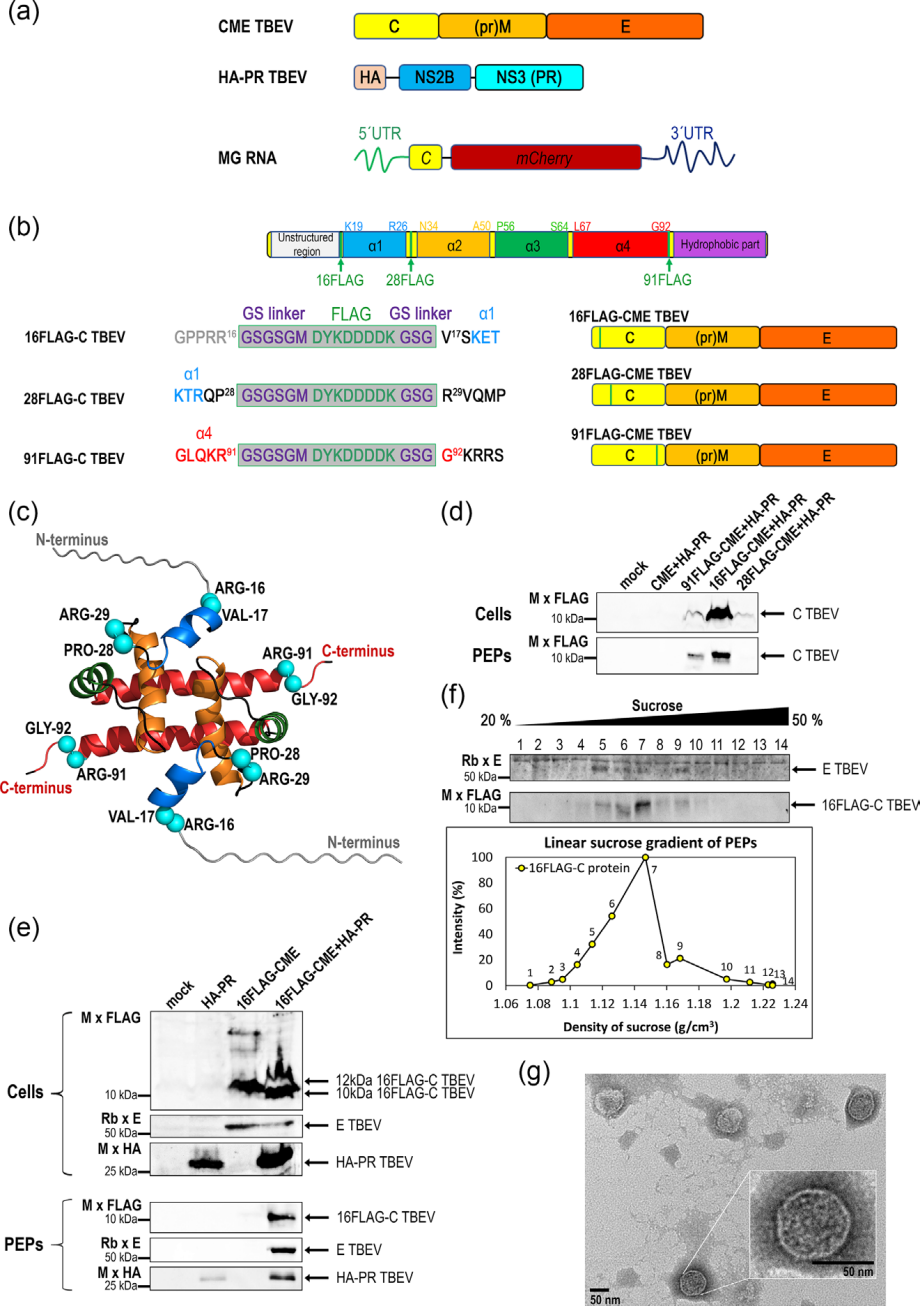
Production and characterization of TBEV VLPs. (**a**) Schematic representation of the TBEV VLP system consisting of three vectors: CME TBEV, encoding the structural polyproteins C (yellow), prM (orange) and E (dark orange); HA-PR TBEV, encoding the viral protease NS2B (blue) and NS3 (turquoise) with an N-terminal HA tag (beige); and MG RNA, encoding a TBEV-derived minigenome containing the 5′ UTR (green), the first 20 codons of the C gene (yellow), the mCherry reporter (red) and the 3′ UTR (dark blue). (**b**) Schematic and amino acid representation of FLAG (green line) insertions within the C protein. Individual helices are shown in distinct colours: α1 (blue), α2 (orange), α3 (green) and α4 (red), consistent with the colour coding published by Selinger *et al*. [43]. The FLAG epitope (DYKDDDDK) was introduced via a flexible glycine–serine (GS) linker between Arg16 and Val17, Pro28 and Arg29 and Arg91 and Gly92 to generate the corresponding vectors 16FLAG-CME TBEV, 28FLAG-CME TBEV and 91FLAG-CME TBEV. (**c**) Schematic representation of the TBEV C dimer with FLAG insertion sites based on the NMR structure (PDB ID: 7YWQ). The truncated unstructured N-terminus (residues 1–16) was modelled in PyMOL. The individual helices are colour-coded as in (**b**). The amino acids flanking the FLAG insertion sites are shown as turquoise spheres. (**d**) Immunochemical detection of FLAG-C protein in cell lysates (Cells) and PEPs obtained from culture media after ultracentrifugation through a 20% sucrose cushion from HEK 293 cells transfected with HA-PR and individual FLAG-CME constructs. (**e**) Immunochemical detection of 16FLAG-C, E and HA-NS2B-NS3 proteins in cell lysates (Cells) and PEPs obtained after ultracentrifugation through a 20% sucrose cushion. (**f**) Linear sucrose (20–50%) gradient fractionation of PEPs. Upper panel: immunoblot analysis showing the distribution of 16FLAG-C and E proteins across gradient fractions. Lower panel: quantitative image analysis of 16FLAG-C signal, expressed as relative band intensity across individual gradient fractions (dots represent values from individual fractions). Fractions 5–8 correspond to a sucrose density of 1.11–1.16 g cm^−^³. (**g**) Transmission electron micrographs of negatively stained TBEV VLPs obtained from sucrose gradient fractions 5–8 shown in (**f**). Scale bar: 50 nm.

The assembly of TBEV is initiated by the interaction between the C protein and gRNA, leading to the formation of a nucleoprotein complex. However, the precise molecular mechanisms of these C–gRNA interactions remain poorly understood. To investigate determinants of gRNA encapsidation by the TBEV C protein, we developed a VLP system based on a previous study by Tang *et al.* [39]. This system comprises three essential TBEV components: (i) a polyprotein (C/prM/E, CME) containing all structural proteins, (ii) a viral protease NS2B-NS3 (HA-PR) and (iii) a TBEV-gRNA-derived minigenome (MG RNA) (Fig. 1a). All components were expressed using the vector driven by the CMV promoter. Co-transfection of HEK 293 cells with CME, HA-PR and MG vectors resulted in ~90% cell viability compared with the non-transfected control, indicating minimal cytotoxicity.

To monitor VLP production, we had to deal with the problem that commercial anti-C antibodies were not available and that the custom-made antibodies exhibited a high background. Therefore, guided by its known four-helix structure [43], we engineered a FLAG-epitope (DYKDDDDK) tagged with a GS linker and inserted it into three distinct positions within the C protein (Fig. 1b, c). The FLAG epitope was introduced (i) downstream of Arg16 (16FLAG-C), between the unstructured N-terminus and helix α1; (ii) downstream of Pro28 (28FLAG-C), in the loop between helix α1 and α2; or (iii) downstream of Arg91 (91FLAG-C) at the C-terminus of helix α4. All insertions were introduced in the CME vector generating three corresponding vectors: 16FLAG-CME TBEV, 28FLAG-CME TBEV and 91FLAG-CME TBEV (Fig. 1b). Because viral particle formation depends on proteolytic processing of CME by the TBEV protease NS2B-NS3 (PR), we also engineered an HA-PR TBEV construct, where an HA epitope (YPYDVPDYA) is connected via a short linker (LMAMEARIP) to the N-terminus of the NS2B-NS3 (Fig. 1a). To assess the production of all FLAG-CME constructs, we performed immunoblotting on samples collected from transfected cells and culture medium (Fig. 1d). This analysis confirmed the specificity of the monoclonal anti-FLAG antibody used and revealed that the 16FLAG-CME construct yielded the highest levels of C protein expression and release of pelletable extracellular particles (Fig. 1d). This construct, therefore, was selected for subsequent experiments. To analyse the proper processing of 16FLAG-CME and release of extracellular particles into the culture medium, HEK 293 cells were transfected with 16FLAG-CME and HA-PR vectors, either individually or in combination. Immunoblot analysis of cells co-transfected with both vectors detected 16FLAG-C, E and HA-PR proteins at expected molecular weights (Fig. 1e, Cells). In cells transfected with a single vector, HA-PR was detected at the expected molecular weight of 34 kDa. In contrast, the 16FLAG-CME vector produced the 74 kDa CME polyprotein but was also partially processed, likely by cellular proteases, yielding immature 12 kDa 16FLAG-C protein and the properly cleaved 54 kDa E protein (Fig. 1e, Cells). The culture media of all the transfected cells were concentrated by ultracentrifugation through a 20% sucrose cushion and analysed immunochemically (Fig. 1e, PEPs). TBEV C and E proteins were detected in the PEPs only when both vectors were co-expressed. Notably, HA-PR was also detected in the PEPs in the absence of CME, which is consistent with previous findings reporting the association of flaviviral proteins with EVs [30].

To further characterize the particle uniformity and morphology, the concentrated culture medium from the cells co-transfected with the 16FLAG-CME and HA-PR vectors was subjected to a 20–50% linear sucrose gradient, and following ultracentrifugation, the individual fractions were analysed immunochemically (Fig. 1f, upper panel). Image analysis of the immunoblot confirmed that extracellular particles, including VLPs, were concentrated within fractions 5–8, corresponding to a sucrose density of 1.11–1.16 g cm^−3^ (Fig. 1f, lower panel), which is consistent with the values reported by Tang *et al*. [39]. TEM analyses of these fractions revealed predominantly uniform spherical particles with a diameter of ~50 nm (Fig. 1g), which corresponds to authentic TBEV virions. However, occasional heterogeneity was also observed (Fig. S1).

### The effect of truncations of MG RNA on the RNA incorporation into TBEV extracellular particles

**Fig. 2.**
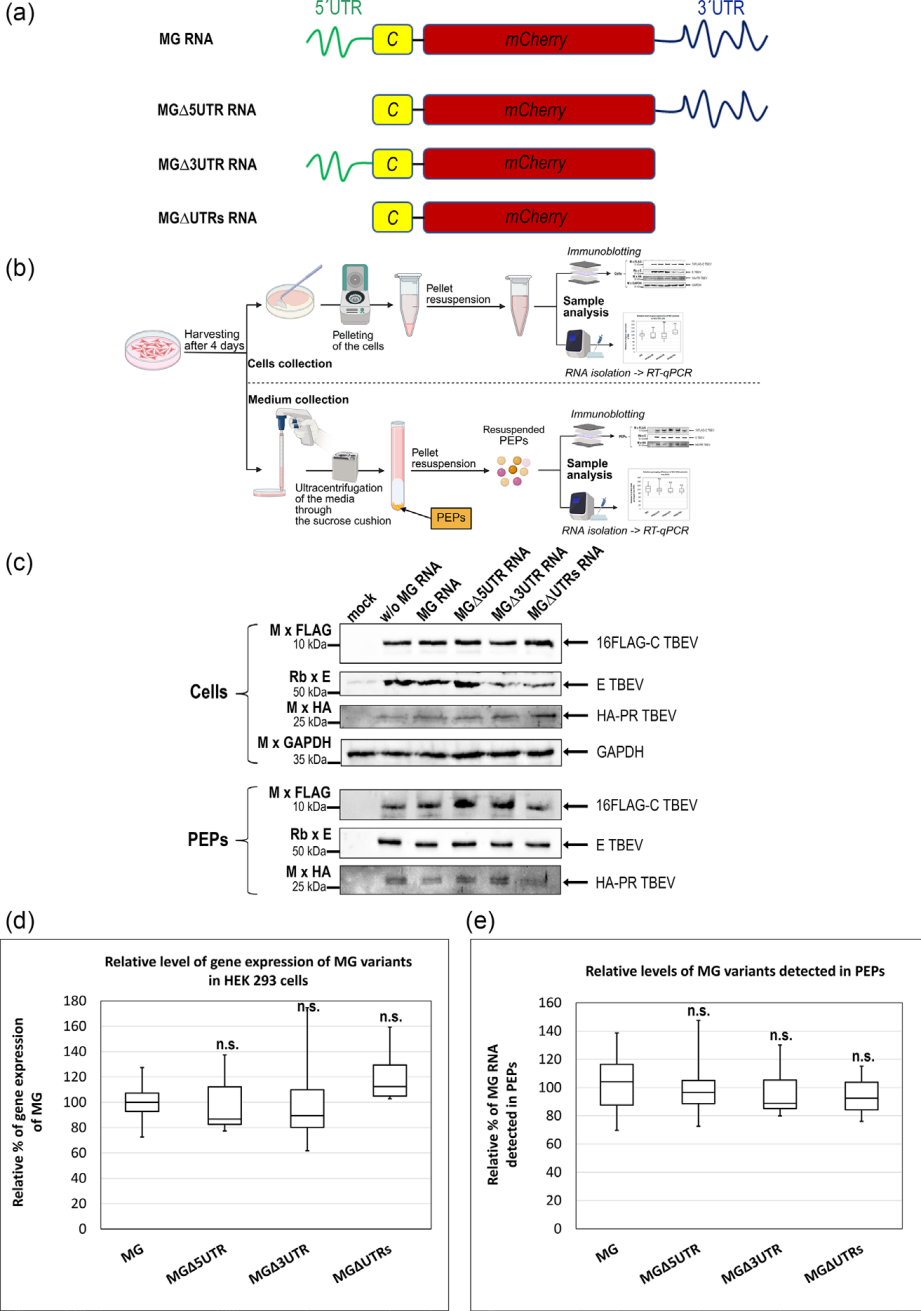
The effect of TBEV minigenome MG truncations on RNA incorporation into PEPs. (**a**) Schematic representation of the full-length MG construct and its truncated variants lacking the 5′ UTR (Δ5UTR), 3′ UTR (Δ3UTR) or both UTRs (ΔUTRs). The full-length MG encodes the 5′ UTR (green), the first 20 codons of the C gene (yellow), the mCherry reporter (red) and the 3′ UTR (dark blue). (**b**) Schematic overview of the experimental workflow used for analysis of MG expression and incorporation. HEK 293 cells were harvested 4 days post-transfection for analysis of intracellular protein and RNA levels (upper panel). Culture supernatants were collected, ultracentrifuged through a 20% sucrose cushion to PEPs and subsequently analysed for extracellular particle-associated proteins and RNA (lower panel). (**c**) Immunoblot analysis of cell lysates (Cells) and PEPs obtained by ultracentrifugation through a 20% sucrose cushion from HEK 293 cells transfected with 16FLAG-CME, HA-PR and the indicated MG variants. Viral proteins were detected using antibodies against FLAG (C protein), E protein and HA (NS2B-NS3). GAPDH served as a loading control for cell lysates. (**d**) Total RNA was extracted from HEK 293 cells transfected with 16FLAG-CME, HA-PR and indicated MG variants, followed by reverse transcription and qPCR using mCherry-specific primers. Intracellular RNA levels of MG variants are shown relative to the full-length MG. (**e**) HEK 293 cells were transfected with 16FLAG-CME, HA-PR and the indicated MG variants. After 4 days, the medium was harvested and ultracentrifuged, and the incorporated RNA was extracted from PEPs. Following reverse transcription, qPCR was performed using mCherry-specific primers. MG RNA levels detected in extracellular particles were normalized to the corresponding intracellular MG RNA levels and expressed relative to the full-length MG. Data are shown as box-and-whisker plots representing three independent biological replicates, each analysed in four technical replicates (*n*=12). Boxes indicate the interquartile range with the median, and whiskers represent the minimum and maximum values. Statistical analysis was performed using ANOVA followed by the Tukey–Kramer HSD test and the Scheffé, Bonferroni and Holm multiple comparison tests. Differences were assessed as not significant (n.s.) with *P*>0.05 compared to the full-length MG.

Most studies suggest that C–gRNA interactions are largely nonspecific, driven predominantly by electrostatic interactions between the negatively charged gRNA and the positively charged interface of the C protein dimer, formed by the α4–α4´ helices. Nevertheless, several reports have indicated a degree of specificity, potentially mediated by stem-loop structures within UTRs [28,29, 44,47]. To examine possible preferences for either the 5′ UTR or 3′ UTR, we constructed MG RNA consisting of the 5′ UTR, the first 20 codons of the C genes, the mCherry reporter and the 3′ UTR, together with three MG-truncated variants lacking either the 5′ UTR (∆5UTR), 3′ UTR (∆3UTR) or both regions (∆UTRs) (Fig. 2a). These constructs were co-transfected with 16FLAG-CME and HA-PR vectors and analysed according to the experimental scheme shown in Fig. 2(b). Levels of MG RNA in transfected cells and PEPs were quantified by qPCR. In parallel, the presence of TBEV proteins in the cell lysates and in PEPs from the culture medium was analysed immunochemically (Fig. 2c).

The expression of TBEV proteins and their presence in PEPs were not affected by the co-expression of the MG vector variants, as verified by immunodetection (Fig. 2c). qPCR analysis of RNA extracted from the transfected cells revealed comparable intracellular levels of MG and MG-truncated variant transcripts, indicating that deletions within the UTRs did not affect RNA synthesis or stability (Fig. 2d). Similarly, analysis of MG RNA detected in PEPs, normalized to intracellular MG levels, showed that all variants, ∆5UTR, ∆3UTR and ∆UTRs, were present at comparable levels (Fig. 2e). These results suggested that the C protein does not display a detectable preference for either the 5′ or 3′ UTR during RNA encapsidation into extracellular particles. Moreover, the comparable levels of MG and MG∆UTRs RNA detected in PEPs indicated that RNA incorporation by the C protein occurs in a largely non-specific manner (Fig. 2e). MG constructs are summarized in Table 1.

**Table 1.** Summary of mutations introduced into the TBEV minigenome and C protein and their effects on RNA incorporation

Construct name	Location	Mutation	Expected effect	Observed phenotype
**MG∆5UTR RNA**	RNA	Deletion of 5′ UTR	**Either no effect or reduction** of MG RNA incorporation	Similar to MG RNA ->**no effect**
**MG∆3UTR RNA**	RNA	Deletion of 3′ UTR	**Either no effect or reduction** of MG RNA incorporation	Similar to MG RNA ->**no effect**
**MG∆UTRs RNA**	RNA	Deletion of both UTRs	**Either no effect or reduction** of MG RNA incorporation	Similar to MG RNA ->**no effect**
**H2 16FLAG-C TBEV**	Helix α2 of TBEV C protein	Point substitution of five selected amino acid residues for alanines	Predicted alteration of the C protein dimer interface and/or membrane association, potentially affecting RNA incorporation	**2–3-fold increase in** MG RNA level detected in PEPs compared to wild-type
**H4 16FLAG-C TBEV**	Helix α4 TBEV C protein	Point substitution of eight selected amino acid residues for alanines	Predicted alteration of C-RNA interactions at the dimer interface, potentially reducing MG RNA incorporation	**1.5-fold increase in** MG RNA level detected in PEPs compared to wild-type
**H2+H4 16FLAG-C TBEV**	Helix α2 and α4 of TBEV C protein	Point substitutions combining substitutions of helices α2 and α4	Predicted strong disruption of C protein dimerization, potentially affecting MG RNA incorporation	**2–3-fold increase in** MG RNA level detected in PEPs compared to wild-type

### The impact of amino acid substitutions in the TBEV C protein dimer interface on RNA incorporation

**Fig. 3.**
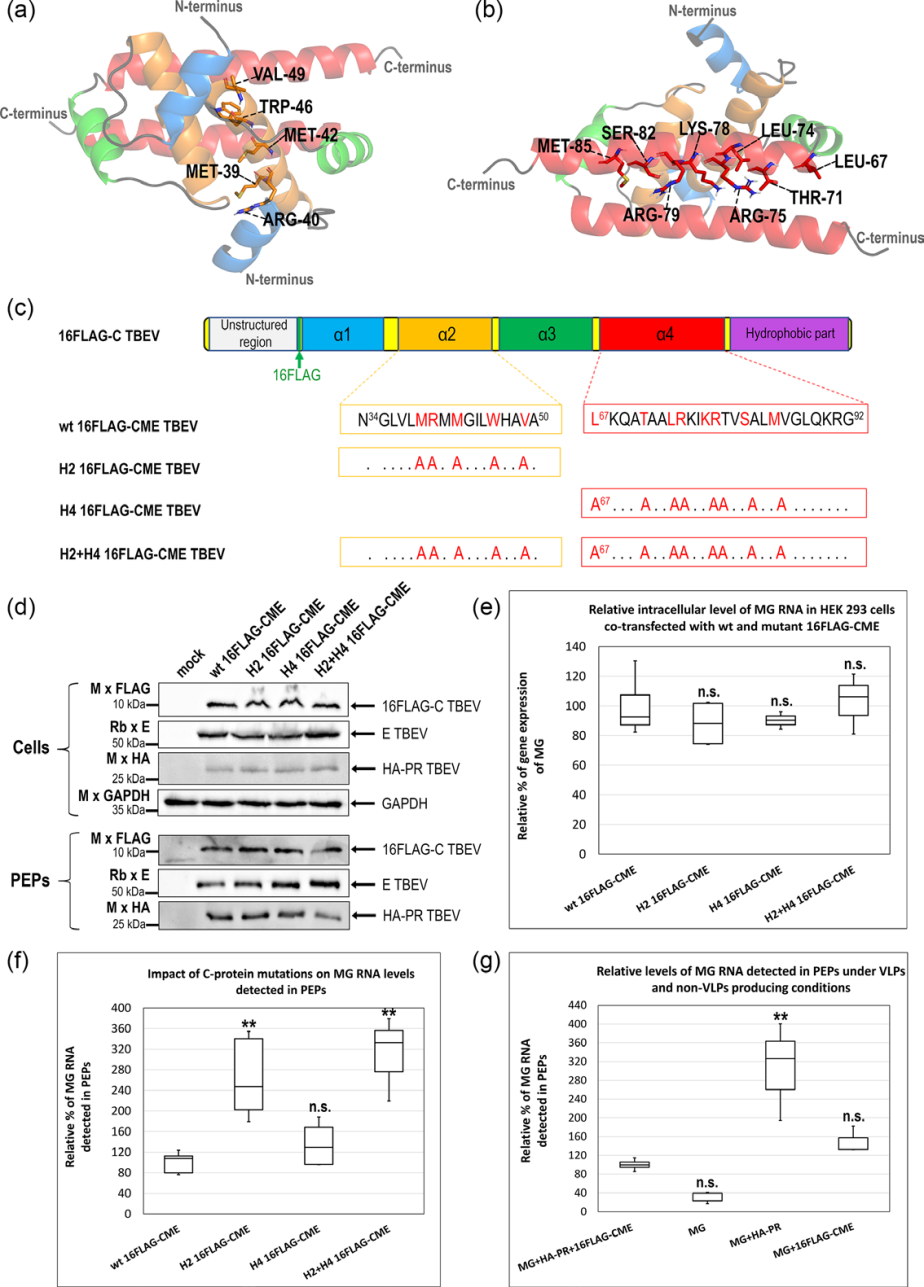
Impact of amino acid substitutions in TBEV C-protein dimer interfaces on RNA incorporation. (**a, b**) Structural model of the TBEV C protein homodimer based on the experimentally determined NMR structure (PDB ID: 7YWQ), highlighting the α2–α2′ interface (top view, **a**) and the α4–α4′ interface (bottom view, **b**) involved in dimerization. Individual helices are colour-coded: α1 (blue), α2 (orange), α3 (green) and α4 (red), consistent with the colour coding used by Selinger *et al*. [43]. Amino acid residues selected for alanine substitution are indicated. (**c**) Schematic representation of the C region of 16FLAG-CME constructs carrying alanine substitution within helix α2 (H2), α4 (H4) or both helices (H2+H4). The substituted amino acid residues are shown in red. All constructs contain a FLAG epitope inserted between residues Arg16 and Val17 of the C protein. (**d**) Immunoblot analysis of cell lysates (Cells) and PEPs obtained after ultracentrifugation through a 20% sucrose cushion from HEK 293 cells transfected with wild-type or mutant (H2, H4 and H2+H4) 16FLAG-CME constructs together with HA-PR and MG RNA. Viral proteins were detected using antibodies against FLAG (C protein), E protein and HA (NS2B-NS3). GAPDH served as a loading control for cell lysates. (**e**) Relative intracellular levels of MG RNA measured in HEK 293 cells co-transfected with wild-type or mutant (H2, H4 and H2+H4) 16FLAG-CME constructs together with HA-PR and MG RNA. Total cellular RNA was isolated, reverse-transcribed and quantified by qPCR using mCherry-specific primers. MG RNA levels are shown relative to the wild-type 16FLAG-C condition. (**f**) Quantification of MG RNA detected in PEPs. HEK 293 cells were transfected with wild-type or mutant 16FLAG-CME constructs together with HA-PR and MG RNA. Total RNA was extracted from cell lysates and from PEPs following ultracentrifugation through a 20% sucrose cushion. After reverse transcription, qPCR was performed using mCherry-specific primers. MG RNA levels detected in extracellular particles were normalized to intracellular MG RNA levels (panel e) and expressed relative to wild-type 16FLAG-C. (**g**) Analysis of extracellular MG RNA detected in the absence of structural or protease viral components. HEK 293 cells were transfected with the indicated combinations of MG RNA, HA-PR and/or 16FLAG-CME constructs. Culture supernatants were ultracentrifuged through a 20% sucrose cushion, and RNA was extracted from cell lysate and obtained pellets. Following reverse transcription, qPCR was performed using mCherry-specific primers. MG RNA levels in extracellular pellets were normalized to intracellular MG RNA levels and expressed relative to the MG+HA-PR+16FLAG-CME condition. Data are shown as box-and-whisker plots representing two independent biological replicates, each analysed in four technical replicates (*n*=8). Boxes indicate the interquartile range with the median, and whiskers represent the minimum and maximum values. Statistical analysis was performed using one-way ANOVA followed by the Tukey–Kramer HSD test and the Scheffé, Bonferroni and Holm multiple comparison tests. Statistical significance is indicated as ** (*P*<0.01) and not significant as n.s. (*P*>0.05) relative to wt 16FLAG-C (**e, f**) or MG+HA-PR+16FLAG-CME (**g**).

It is generally assumed that incorporation of flaviviral gRNA by the C protein is preceded by C protein dimerization, which is likely triggered by proteolytic cleavage of the C protein from viral polyprotein by the viral protease. In a previous study, hydrogen-deuterium exchange analysis revealed that TBEV C protein dimerization is mediated and stabilized primarily by amino acid residues located within the helices α2 (orange in Fig. 3a) and α4 (red in Fig. 3b) [43]. To determine whether disruption of these dimer interfaces affects viral RNA incorporation, we introduced alanine substitutions into the C protein to generate three variants in the 16FLAG CME vector: (i) H2 16FLAG-C TBEV carrying substitutions M39, R40, M42, W46 and V49 in helix α2; (ii) H4 16FLAG-C TBEV containing substitutions L67, T71, L74, R75, K78, R79, S82 and M85 in helix α4; and (iii) H2+H4 16FLAG-C TBEV combining mutations in both α2 and α4 helices as schematically illustrated in Fig. 3(c).

The wild-type and mutant 16FLAG-CME constructs were co-transfected with HA-PR and MG into HEK 293 cells. For simplicity, the C variants are referred to as 16FLAG-C, although they were expressed from the 16FLAG-CME constructs. Immunoblot analysis of cell lysates and PEPs obtained after ultracentrifugation through a 20% sucrose cushion revealed that all C protein variants were expressed and released from the cells at levels comparable to the wild-type construct (Fig. 3d). Consistently, no substantial differences in intracellular MG RNA levels were detected between cells expressing wild-type or mutant 16FLAG-CME (Fig. 3e). Quantitative analysis of MG RNA detected in pelleted extracellular fractions revealed that alanine substitutions within the C protein dimer interfaces resulted in increased levels of MG RNA compared to the wild-type construct (Fig. 3f). The H2 mutant exhibited approximately a 2–3-fold increase in MG RNA detected in PEPs, whereas the H4 mutant showed a more modest (~1.5-fold) increase. The H2+H4 double mutant displayed MG RNA levels comparable to those of the H2 variant (Fig. 3f). These results indicated that partial disruption of the α2 and α4 dimerization interfaces leads to increased levels of MG RNA detected in PEPs under VLP-producing conditions. To determine whether elevated MG RNA levels in PEPs reflect enhanced VLP formation or whether MG RNA can also be detected in the absence of components required for VLP formation, cells were transfected with plasmid combinations lacking either the capsid (16FLAG-CME) or the viral protease (HA-PR) (Fig. 3g). Although both these components are essential for VLP formation, levels of extracellular MG RNA increased in their absence. In particular, production of the viral protease alone (MG+HA PR) resulted in an approximately threefold increase in MG RNA levels in PEPs compared to the complete VLP-forming condition (MG+HA-PR+16FLAG-CME) (Fig. 3g). Because pelleted extracellular fractions were collected by ultracentrifugation through a 20% sucrose cushion, which sediments not only VLPs but also other extracellular pelletable particles, these findings suggest that MG RNA can be released from cells via non-VLP pathways, such as EVs. C protein mutations are summarized in Table 1.

### Incorporation of TBEV proteins and RNA into EVs

Although the role of EVs in the flaviviral replication cycle remains incompletely understood, several studies have reported the presence of viral proteins and RNA in EVs released from cells infected with mosquito-borne viruses, including DENV, ZIKV and WNV [32,33, 37, 48,50]. In contrast, evidence for the involvement of EVs in infections with tick-borne flaviviruses is limited; viral RNA, as well as E and NS1 proteins, has been detected in EVs during LGTV infection, whereas incorporation of TBEV NS2B-NS3 protease into EVs has not yet been reported [30,34].

**Fig. 4.**
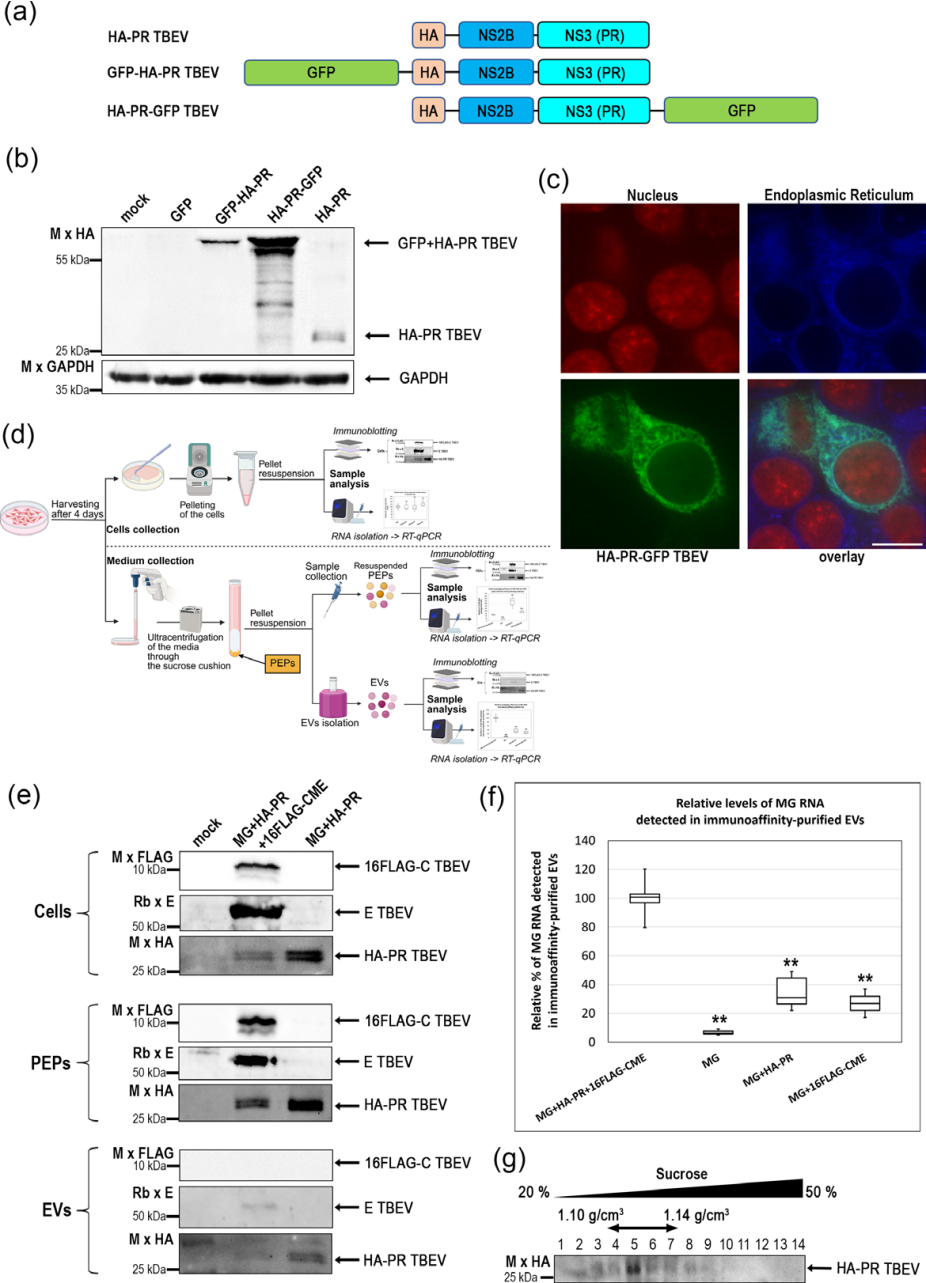
Incorporation of TBEV proteins and RNA into EVs. (**a**) Schematic representation of HA-tagged NS2B-NS3 TBEV protease (HA-PR) fusion constructs used in this study. GFP (green) was fused either to the N-terminus (GFP-HA-PR) or to the C-terminus (HA-PR-GFP). Individual protein domains are indicated: HA tag (beige), NS2B (blue) and the NS3 protease domain (turquoise). (**b**) Immunoblot analysis of HEK 293 cell lysates expressing GFP-HA-PR, HA-PR-GFP or untagged HA-PR, as indicated. Proteins were detected using an antibody against the HA tag. (**c**) Confocal fluorescence microscopy of HA-PR-GFP (green) expressed in mScarlet DHX15 HEK 293 cells. The nucleus is visualized by mScarlet DHX15 (red), and the ER is stained using an ER-Tracker Blue-White DPX (blue). The merged image (overlay) shows partial spatial association of HA-PR-GFP with ER structures. Scale bar represents 10 µm. (**d**) Schematic overview of the experimental workflow used for analysis of extracellular viral components. HEK 293 cells were harvested 4 days post-transfection for analysis of intracellular proteins and RNA (upper part). Culture supernatants were collected and ultracentrifuged through a 20% sucrose cushion to pellet extracellular material containing VLPs and EVs (middle part). Where indicated, EVs were further purified from the pelleted material by immunoaffinity isolation using magnetic beads coated with antibodies against exosomal markers CD9, CD63 and CD81 (lower part). (**e**) Immunoblot analysis of samples prepared as outlined in (**d**). Cell lysates (Cells), pelleted extracellular material containing VLPs and EVs (PEPs) and immunoaffinity-purified EVs (EVs) from HEK 293 cells transfected with the indicated combinations of MG RNA, HA-PR and 16FLAG-CME were analysed. Viral proteins were detected using antibodies against FLAG (C protein), E protein and HA (NS2B-NS3). (**f**) Quantification of MG RNA detected in immunoaffinity-purified EVs. EV samples were isolated as outlined in (**d**) from HEK 293 cells transfected with the indicated vector combinations. Total RNA was extracted from purified EVs, reverse-transcribed and analysed by qPCR using mCherry-specific primers. MG RNA levels were normalized to intracellular MG RNA levels and expressed relative to the MG+HA-PR+16FLAG-CME condition. Data are shown as box-and-whisker plots, where the boxes indicate the median, and the whiskers represent the minimum and maximum values. The data represent two independent biological replicates, each analysed in six technical replicates (*n*=12). Statistical analysis was performed by ANOVA followed by the Tukey–Kramer HSD test and the Scheffé, Bonferroni and Holm multiple comparison tests. Statistical significance is indicated as (**P<0.01) relative to MG+HA-PR+16FLAG-CME. (**g**) Sucrose density gradient (20–50%) analysis of immunoaffinity-purified EVs released from HEK 293 cells transfected with HA-PR and MG RNA. Gradient fractions were analysed by immunoblotting using an anti-HA antibody. HA-PR was detected predominantly in fractions 4–7, corresponding to densities of 1.10–1.14 g cm^−^³.

To further examine our observation that the TBEV NS2B-NS3 protease complex lacking its hydrophobic domain can be released independently of VLP-forming conditions, we expressed HA-NS2B-NS3 fused to GFP at either the N-terminus (GFP-HA-NS2B-NS3; GFP-HA-PR) or the C-terminus (HA-NS2B-NS3-GFP; HA-PR-GFP) (Fig. 4a). Culture supernatants were collected 4 days post-transfection and subjected to ultracentrifugation through a 20% sucrose cushion, and the resulting pellets were analysed by immunoblotting (Fig. 4b). The result showed that HA-PR-GFP was released into the extracellular fraction with higher efficiency than GFP-HA-PR (Fig. 4b). Confocal microscopy revealed that HA-PR-GFP (green) localized in close proximity to the ER (blue) (Fig. 4c), consistent with an association with intracellular membrane compartments involved in EV biogenesis. Colocalization of the green and blue channels was quantified, yielding an average Pearson‘s correlation coefficient of 0.67 (sd, 0.15).

To distinguish EVs from VLPs within PEPs obtained by ultracentrifugation through a 20% sucrose cushion, which contains a mixture of both types of particles, we established an immunoaffinity-based EV isolation workflow using antibodies against the exosomal markers CD9, CD63 and CD81 (schematically illustrated in Fig. 4d). HEK 293 cells were transfected either with MG, HA-PR and 16FLAG-CME to allow VLP formation or with MG and HA-PR only, which does not support VLP assembly. Cell lysates (Cell), PEPs and immunoaffinity-purified EV fractions (EVs) were analysed immunochemically for the presence of C, E and HA-PR proteins (Fig. 4e). As expected, pelleted extracellular material from cells transfected with MG, HA-PR and 16FLAG-CME contained C and E proteins, confirming VLP formation; however, HA-PR was also detected in this fraction (Fig. 4e, PEPs). In cells transfected with MG and HA-PR alone, HA-PR was likewise detected in the pelleted extracellular material (Fig. 4e, PEPs). Importantly, following immunoaffinity purification, the C protein was not detected in EV fractions from either condition (Fig. 4e, EVs), confirming the specificity of EV isolation. In contrast, weak signals corresponding to E and HA-PR were detected in EV fractions from cells expressing MG+HA-PR+16FLAG-CME, while HA-PR was detected in EVs from the cells expressing MG+HA PR alone, indicating partial association of these viral proteins with EVs (Fig. 4e, EVs).

To determine whether EVs also contain viral RNA, immunoaffinity-purified EVs were isolated from HEK 293 cells transfected with different combinations of MG, HA-PR and 16FLAG-CME. MG RNA was detectable in EV fractions under all tested conditions (Fig. 4f). However, MG RNA levels were reduced in EVs isolated from cells lacking HA-PR or 16FLAG-CME compared to the complete VLP-forming condition, indicating that the presence of multiple flaviviral components is associated with increased levels of MG RNA in EVs.

Together, these results demonstrate that TBEV proteins and MG RNA can be detected in EVs not only during infection, as reported previously, but also under conditions supporting VLP formation. Further analysis revealed that immunoaffinity-purified EVs co-fractionate with VLPs upon sucrose density gradient separation (Fig. 4g). Specifically, EVs containing HA-PR were detected predominantly in fractions 4–7, corresponding to sucrose densities of 1.10–1.14 g cm^−3^, which overlap with the same density range of TBEV VLPs (Fig. 1f). This overlap underscores the difficulty of full separation of EVs from VLPs using conventional ultracentrifugation-based approaches and highlights the importance of considering EV-associated release when interpreting VLP-based analyses.

## Discussion

In this study, we developed a TBEV VLP system to characterize C–RNA interactions as an early step in flavivirus assembly. This system is based on three plasmids that encode the structural polyprotein (16FLAG-C/prM/E), the viral protease (HA-NS2B/NS3) and the TBEV-gRNA-derived minigenome (MG RNA) (Fig. 1a). It enables the flexible manipulation of both the protein and RNA components and allows the quantitative analysis of RNA incorporation. Using this platform, we addressed two fundamental questions: (i) whether the 5′ and 3′ UTRs contribute to the selective packaging of the viral genome and (ii) whether disruption of the dimerization interface in the C protein affects RNA incorporation.

A key technical advancement in our study was the introduction of FLAG-tagged capsid variants, which overcame the lack of reliable anti-C antibodies. Previous studies primarily relied on the detection of the E protein [39,51], the fusion of the C protein to large reporter proteins such as mCherry or MBP [52,53] or the insertion of a 3×FLAG tag [54]. However, because flaviviral prM and E proteins can be released in the form of subviral particles lacking nucleocapsids, detection of the E protein alone does not provide reliable information about RNA-containing particles [55]. Guided by our NMR structure of the TBEV C protein [43] and structural modelling (Fig. 1c), we systematically tested three FLAG insertion sites and identified the 16FLAG construct as optimal (Fig. 1b–d). This construct provided efficient expression and release of pelletable extracellular particles and is consistent with structural predictions that insertion at this position does not disrupt C protein dimerization or interactions with RNA or membranes. Immunoblot analyses confirmed proper processing of the 16FLAG-CME polyprotein by the HA-NS2B-NS3 protease, in agreement with the established sequential cleavage model of flaviviral polyprotein processing (Fig. 1e). Interestingly, cleavage of the E protein was also detected in the absence of the viral protease, which is consistent with earlier reports that host proteases can process E protein during VLP production [56]. The presence of VLPs in pelleted extracellular material was further supported by linear sucrose density gradient and TEM (Fig. 1f, g). In agreement with the previous study [39], VLPs were concentrated at densities of 1.11–1.16 g cm^−3^ and displayed predominantly uniform morphology, although some size heterogeneity was observed (Fig. S1).

Using this VLP system, we first assessed the contribution of the 5′ and 3′ UTRs to RNA incorporation (Table 1). Deletion of the 5′ UTR, the 3′ UTR or both regions did not significantly affect the levels of MG RNA detected in PEPs (Fig. 2d, e), indicating that these regions are not essential for RNA incorporation under VLP-producing conditions. Although previous studies proposed that RNA secondary structures within UTRs may contribute to packaging specificity [45,47], either directly or through interactions with non-structural proteins, such as NS2A [28,29], our data support a model in which C–RNA interactions are largely non-specific and primarily driven by electrostatic interactions. These findings suggest that UTR-dependent specificity is not a dominant determinant of RNA incorporation in the context of VLP assembly.

We next examined the role of capsid dimerization by introducing alanine substitutions into helices α2 and α4, which form the principal dimerization interfaces of the TBEV C protein (Table 1). Contrary to expectations, and in contrast to previous observations by Figueira-Mansur *et al*. for DENV, where similar mutations destabilized C–RNA interactions [57], disruption of these interfaces in TBEV C resulted in increased levels of MG RNA detected in pelleted extracellular fractions (Fig. 3f). The strongest effect was observed for the H2 and combined H2+H4 mutants. One possible explanation is that disruption of native dimer interfaces alters the C protein surface in a manner that promotes non-specific RNA association. This interpretation is supported by our observation that the H2 and H2+H4 variants fail to form stable dimers, with the H2+H4 mutant being predominantly monomeric (Novotný *et al*., unpublished data). In such a scenario, monomeric C proteins may exhibit altered RNA-binding properties or associate with RNA in a less regulated manner, potentially allowing incorporation of multiple copies of the smaller MG RNA.

Importantly, our data also indicate that elevated levels of MG RNA in pelleted extracellular material do not necessarily reflect increased VLP formation. Indeed, MG RNA was detected in pelleted extracellular fractions even in the absence of structural proteins required for VLP assembly (Fig. 3g), indicating the involvement of alternative release pathways. A particularly unexpected finding was the detection of the viral protease NS2B-NS3 in the extracellular environment, even in the absence of structural proteins (Fig. 1e, PEPs). To our knowledge, NS2B-NS3 has not been previously reported in mature flaviviral particles or VLPs. This observation prompted us to investigate the contribution of EVs, as an alternative route for the release of viral components. EV-mediated release of viral proteins and RNA has been documented for several flaviviruses, including DENV, ZIKV, YFV or LGTV [32,38].

EV formation is enhanced during viral replication and is associated with extensive membrane remodelling, particularly at the ER [58,62]. Several ER-associated proteins involved in membrane curvature and remodelling, such as Alastin or Reticulon-3, have been shown to be incorporated into EVs during flaviviral or HCV (hepatitis C virus) infection [63,64] together with various viral components, including RNA, structural proteins and non-structural proteins. The purpose of the secretion of the components via EVs remains unknown; however, several studies demonstrated EVs as an alternative route for transmission of the flaviviral gRNA. EVs released from mosquito and tick cells infected with DENV, ZIKV and LGTV have been shown to infect human cells [32,34, 48, 49]. EV-mediated transmission of HCV was also successful, albeit with lower efficiency compared to the infection with authentic viral particles [65,66]. Consistent with this, our GFP-tagging experiments demonstrated that NS2B-NS3 localizes in close proximity to ER (Fig. 4c), despite lacking a classical membrane anchor. Immunoaffinity isolation of EVs using antibodies against exosomal markers CD9, CD63 and CD81 confirmed the presence of NS2B-NS3 and MG RNA in purified EV fractions (Fig. 4e, f).

MG RNA was also detected in EVs under all tested conditions, including the absence of structural proteins, supporting that RNA can be released independently of VLP formation. Notably, the highest level of MG RNA in EVs was observed under VLP-producing conditions, suggesting that the presence of multiple viral components enhances RNA incorporation into EVs. In addition to NS2B-NS3, the E protein was also detected in EV fractions (Fig. 4e), in agreement with previous studies reporting incorporation of flaviviral E protein into EVs [32,34, 35, 48, 65, 67]. The absence of C protein in purified EVs supports the specificity of the immunoaffinity isolation and argues against contamination by VLPs.

Finally, we demonstrated that EVs containing viral components co-fractionate with VLPs during sucrose density gradient centrifugation (Fig. 4g), which is consistent with previous findings [68,72]. This underscores the difficulty of separating these particle populations using conventional methods. These overlaps have important implications for studies of flaviviral assembly and for the development of VLP-based vaccines, where EVs may represent an unavoidable impurity.

In summary, our study provides several key insights into flaviviral RNA incorporation. We established FLAG-tagged TBEV capsid constructs as robust tools for monitoring the C protein-containing extracellular particles, demonstrated that RNA incorporation occurs largely independently of UTR sequences and showed that disruption of capsid dimer interfaces paradoxically enhances RNA level detected in extracellular fractions. Importantly, we identified EVs as a parallel pathway for the release of viral proteins and RNA, highlighting the necessity of distinguishing between VLPs and EVs in mechanistic studies and vaccine development.

## Supplementary material

10.1099/jgv.0.002258 Uncited Supplementary Material 1.

## Abbreviations

C: capsid
DENV: Dengue virus
E: envelope
ER: endoplasmic reticulum
EV: extracellular vesicle
gRNA: genomic RNA
GS: glycine–serine
HCV: hepatitis C virus
HEK 293 cells: human embryonic kidney 293 cells
JEV: Japanese encephalitis virus
LGTV: Langat virus
PEI: polyethylenimine
PEPs: pelleted extracellular particles
PLB: protein loading buffer
prM: premembrane
qPCR: quantitative PCR
TBEV: tick-borne encephalitis virus
TEM: transmission electron microscopy
VLP: virus-like particle
WNV: West Nile virus
YFV: Yellow fever virus
ZIKV: Zika virus
