# Supplementary material for: Co-release of tick-borne encephalitis virus RNA and structural proteins via virus-like particles and extracellular vesicles
Source: J Gen Virol. 2026 Apr 29;107(4):002258. doi: 10.1099/jgv.0.002258 (PMC13135480; doi:10.1099/jgv.0.002258)
Supplement: Uncited Supplementary Material 1. [file jgv-107-02258-s001.pdf]

Supplementary Table S1. Sequences (5´-3´) of all primers used for cloning and qPCR.

**Introduction of the FLAG tag into the TBEV C protein**	
**TBEV_C16_FLAG_Fw**	ATGGACTACAAAGACGATGACGACAAGGGTTCAGGAGTGTCGAAAGAGACC
**TBEV_C16_FLAG_Rev**	ATCGTCTTTGTAGTCCATTCCACTACCTGAACCTCGTCGAGGGGGACC
**TBEV_C28_FLAG_For**	ATGGACTACAAAGACGATGACGACAAGGGTTCAGGAAGAGTCCAAATGCCAA
**TBEV_C28_FLAG_Rev**	ATCGTCTTTGTAGTCCATTCCACTACCTGAACCGGGTTGACGCGTCTTCG
**TBEV_C91_FLAG_For**	ATGGACTACAAAGACGATGACGACAAGGGTTCAGGAAAAAGGAGGTCAG
**TBEV_C91_FLAG_Rev**	ATCGTCTTTGTAGTCCATTCCACTACCTGAACCGCGTTTTTGCAAGCC
**Introduction of mutations into the helix α2 of the TBEV C protein**	
**pCME-wt/ H2/mut1_Fw**	GTTGGCAGCCATGATGGGGATCTTG
**pCME-wt/ H2/mut1_Rev**	CATGGCTGCCAACACGAGCCCATT
**pCME-wt/H2/mut2_Fw**	CATGGCAGGGATCTTGTGGCAT
**pCME-wt/ H2/mut2_Rev**	CCCTGCCATGCGCATCAACAC
**pCME-wt/ H2/mut3_Fw**	GATCTTGGCACATGCCGTAGCTG
**pCME-wt/ H2/mut3_Rev**	GGCATGTGCCAAGATCCCCATCAT
**pCME-wt/ H2/mut4_Fw**	CATGCCGCAGCTGGCACC
**pCME-wt/ H2/mut4_Rev**	CAGCTGCGGCATGTGCCAAGAT
**pCME-wt/ H2/mut5_Rev**	GATCCCTGCCATGGCTGCCAA
**pCME-wt/ H2/mut6_Rev**	GGCATGTGCCAAGATCCCTGCCAT
**Introduction of mutations into the helix α4 of the TBEV C protein**	
**L67A-TBEVC_Fw**	CAGTCCCTGCAAAACAGGCC
**L67A-TBEVC_Rev**	GGCCTGTTTTGCAGGGACTG
**T71A-TBEVC_Fw**	CAGGCCGCAGCAGC
**T71A-TBEVC_Rev**	GCTGCTGCGGCCTG
**S82A-TBEVC_Fw**	CAGTGGCCGCCC
**S82A-TBEVC_Rev**	GGGCGGCCACTG
**M85A-TBEVC_Fw**	GCCCTAGCCGTTGGCTTG
**M85A-TBEVC_Rev**	CAAGCCAACGGCTAGGGC
**L74A-R75A-TBEVC_Fw**	GCAGCGAAGATCAAAAGGACAGTGAGTGCCC
**L74A-R75A-TBEVC_Rev**	TTTGATCTTCGCTGCTGCTGCTGTGGCC
**K78A-R79A-TBEVC_Fw**	GCCGCAACAGTGAGTGCCCTAATGGTTGG
**K78A-R79A-TBEVC_Rev**	ACTCACTGTTGCGGCGATCTTCCGCAGTG
**Construction of plasmids containing gene encoding NS2B-NS3**	
**5_EcoRI_NS2B**	AAAGAATTCCGTCTTTCAGTGAACCACTAACTGTG
**3_NS2B_KpnI**	TTTGGTACCACCTCCACCAGAACC
**5_KpnI_NS3**	AAAGGTACCTCTGACCTGGTTTTCTCTG
**3_NS3_NotI**	ATTCTTATGCGGCCGCCTATGATGTCCAGCCCGT
**GFP_no_ATG_Fw**	GTGAGCAAGGGCGAGG
**GFP_no_STOP_Rev**	CTTGTACAGCTCGTCCATGC
**GFP_HANS2BNS3_Fw**	GACGAGCTGTACAAGGGATCTGGATACCCATACGATGTTCC
**GFP_HANS2BNS3_Rev**	CTCGCCCTTGCTCACCATGTACAATTCCGCAG
**HANS2BNS3_GFP_Fw**	GGACGAGCTGTACAAGtagGGGATCCAGACATGATAAGATAC
**HANS2BNS3_GFP_Rev**	CTCGCCCTTGCTCACTCCTGATCCAGATCCTGATGTCCAGCCCGTG
**Construction of plasmids containing MG**	
**pUC19_1_Fw**	TCGGTACCCGGGGATCCTCT
**pUC19_1_Rev**	GCTCGAATTCACTGGCCGTC
**5´UTR_T7_Fw**	GACGGCCAGTGAATTCGAGCTAATACGACTCACTATAGGGAG
**5´UTR_T7_mCher-Rev**	CCTTGCTCACTTTCGACACTCGTCGAGG
**mCherry_mini-Fw**	AGTGTCGAAAGTGAGCAAGGGCGAGGAG
**mCherry_mini-Rev**	CAGTCTGGGTTTACTTGTACAGCTCGTCCATG
**3´UTR_ mCher-Fw**	GTACAAGTAAACCCAGACTGTGACAGAG
**3´UTR_Rev**	AGAGGATCCCCGGGTACCGACTCGAGAGCGGGTGTTTTTCCGAG
**5_SacI_5_UTR**	TTTGAGCTCGTGGAGATTTTCTTGC
**3_XhoI_3_UTR**	TTTCTCGAGAGCGGGTGTTTTTCC
**3_mCherry_XhoI**	TTTCTCGAGTTACTTGTACAGCTC
**5_SacI_C_protein**	AAAGAGCTCGGGATGGTCAAGAAGG
**Primers used for qPCR**	
**mCherry_Fw**	CCTGCAGGACGGCGAGTTCATCTA
**mCherry_Rev**	GTGGCCGCCGTCCTTCAGCTTC
**GAPDH_Fw**	GAAGGTGAAGGTGGGAGTC
**GAPDH_Rev**	GAAGATGGTGATGGGATTTC

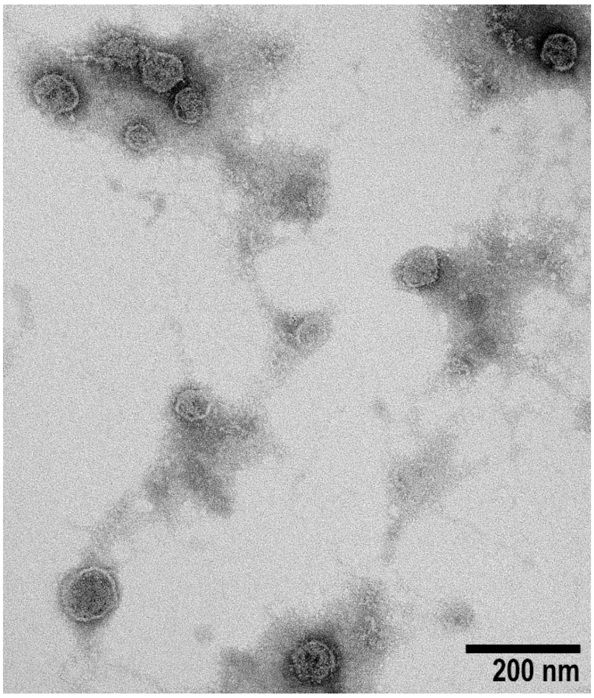

Supplementary Figure S1. Transmission electron micrographs of a negatively stained sample containing particles of different sizes, including TBEV VLPs, released from HEK 293 cells. Scale bar: 200 nm.

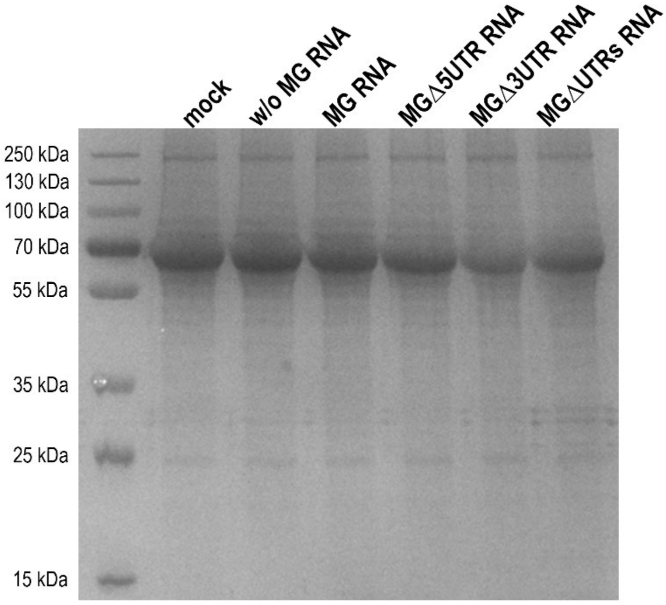

Supplementary Figure S2. Coomassie Brilliant Blue-stained SDS-PAGE gel showing the total protein content of pelleted extracellular particles (PEPs) obtained by ultracentrifugation through a 20% sucrose cushion from culture media of HEK 293 cells transfected with 16FLAG-CME, HA-PR, and the indicated MG variants. The analyzed samples correspond to those used for immunoblot analysis of PEP fractions in Fig. 2C.

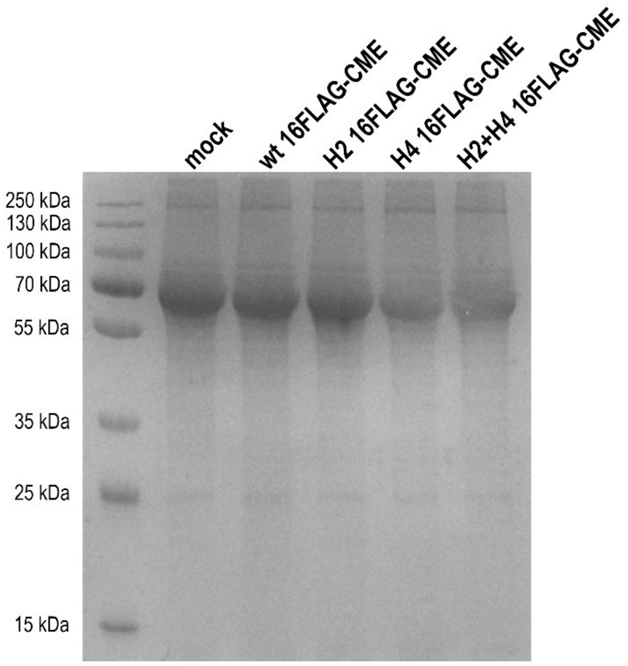

Supplementary Figure S3. Coomassie Brilliant Blue-stained SDS-PAGE gel showing the total protein content of pelleted extracellular particles (PEPs) obtained by ultracentrifugation through a 20% sucrose cushion from culture media of HEK 293 cells transfected with wild-type or mutant 16FLAG-CME constructs together with HA-PR and MG. The analyzed samples correspond to those used for immunoblot analysis of PEP fractions in Fig. 3D.
